# Visceral Adiposity Index and Prognostic Nutritional Index in Predicting Atrial Fibrillation after On-Pump Coronary Artery Bypass Operations: a Prospective Study

**DOI:** 10.21470/1678-9741-2020-0044

**Published:** 2021

**Authors:** Mesut Engin, Kadir Kaan Ozsin, Muhammed Savran, Orhan Guvenc, Senol Yavuz, Ahmet Fatih Ozyazicioglu

**Affiliations:** 1 Department of Cardiovascular Surgery, University of Health Sciences, Mehmet Akif İnan Training and Research Hospital, Karaköprü/Şanlıurfa, Turkey.; 2 Department of Cardiovascular Surgery, University of Health Sciences, Bursa Yuksek Ihtisas Training and Research Hospital, Bursa, Turkey.; 3 Department of Cardiovascular Surgery, Medical Faculty of Uludağ University, Bursa, Turkey.

**Keywords:** Adiposity, Prognosis, Nutritional Assessment, Atrial Fibrillation, Waist Circumference, Lymphocyte Count, Postoperative Term

## Abstract

**Introduction:**

Rhythm problems are the most observed complications following coronary artery bypass grafting (CABG), the most common being postoperative atrial fibrillation (PoAF), with an incidence reaching 50% of the patients. In this study, we aimed to investigate the predictive importance of prognostic nutritional index (PNI) and visceral adiposity index (VAI) in predicting PoAF, which occurs after CABG accompanied by cardiopulmonary bypass.

**Methods:**

Patients who underwent isolated CABG with cardiopulmonary bypass between June 15 and October 15, 2019, were prospectively included in the study. Patients who did not develop in-hospital PoAF were identified as Group 1, and those who did constituted Group 2.

**Results:**

PoAF developed in 55 (27.6%) patients (Group 2). The mean age of the 144 patients included in Group 1 and 55 patients in Group 2 were 56.9±8.7 and 64.3±10.2 years, respectively (*P*<0.001). In multivariate analysis Model 1, age (odds ratio [OR]: 1.084, confidence interval [CI]: 1.010-1.176, *P*=0.009), chronic obstructive pulmonary disease (OR: 0.798, CI: 0.664-0.928, *P*=0.048), and PNI (OR: 1.052, CI: 1.015-1.379, *P*=0.011) were determined as independent predictors for PoAF. In Model 2, age (OR: 1.078, CI: 1.008-1.194, *P*=0.012), lymphocyte counts (OR: 0.412, CI: 0.374-0.778, *P*=0.032), and VAI (OR: 1.516, CI: 1.314-2.154, *P*<0.001) were determined as independent predictors for PoAF.

**Conclusion:**

In this study, we determined that low PNI, a simply calculable and cheap parameter, along with high VAI were risk factors for PoAF.

**Table:** 

Abbreviations, acronyms & symbols				
**ACE-I**	**= Angiotensin-converting enzyme inhibitor**		**HDL-C**	**= High-density lipoprotein cholesterol**
**AF**	**= Atrial fibrillation**		**HT**	**= Hypertension**
**ARB**	**= Angiotensin receptor blocker**		**ICU**	**= Intensive care unit**
**AUC**	**= Areas under the curve**		**LDL-C**	**= Low-density lipoprotein cholesterol**
**BMI**	**= Body mass index**		**OR**	**= Odds ratio**
**CABG**	**= Coronary artery bypass grafting**		**PCI**	**= Percutaneous coronary intervention**
**CCt**	**= Cross-clamp time**		**PNI**	**= Prognostic nutritional index**
**CI**	**= Confidence interval**		**PoAF**	**= Postoperative atrial fibrillation**
**COPD**	**= Chronic obstructive pulmonary disease**		**ROC**	**= Receiver-operating characteristic**
**CPB**	**= Cardiopulmonary bypass**		**SD**	**= Standard deviation**
**CRP**	**= C-reactive protein**		**TG**	**= Triglycerides**
**ECG**	**= Electrocardiography**		**TPt**	**= Total perfusion time**
**EuroSCORE II**	**= European System for Cardiac Operative Risk Evaluation II**		**TSH**	**= Thyroid-stimulating hormone**
			**VAI**	**= Visceral adiposity index**
**HDL**	**= High-density lipoprotein**		**WC**	**= Waist circumference**

## INTRODUCTION

Coronary artery bypass grafting (CABG) is a major surgery; hence, it should be performed with particular care and discipline. Combating postoperative complications is as important as surgical technique. Rhythm problems are the most observed complications following CABG, the most common being postoperative atrial fibrillation (PoAF), with an incidence reaching 50% of the patients. Known risk factors for this condition are advanced age, low ejection fraction, chronic obstructive pulmonary disease (COPD), and thyroid hormone disorders^[^^1^^]^. A recent study has shown that it increases hospital costs by up to three times, and causes significant morbidity, such as thromboembolic problems and prolonged hospital admissions^[^^2^^]^.

Obesity is one of the most important problems of our era. High body mass index (BMI) has been shown as a risk factor in many studies in terms of its effects on outcomes after coronary surgery. It is known that increased BMI increases PoAF^[^^3^^]^. However, among cardiovascular risk factors, the usability of BMI is controversial, which is defined as the “obesity paradox”^[^^4^^]^. Abdominal obesity is thought to be a better predictor for cardiovascular diseases, a valuable indicator for it is the visceral adiposity index (VAI), found to be more sensitive and specific in terms of cardiovascular risk compared to BMI^[^^5^^]^. Therefore, VAI may be a risk factor for PoAF.

Prognostic nutritional index (PNI) is a parameter that can be calculated by albumin and lymphocyte count. The importance of albumin stems from its anti-inflammatory, antioxidant, and anticoagulant properties and its effects on osmotic pressure. Lymphopenia is associated with major adverse events in atherosclerotic cardiovascular diseases. Neutrophilia is induced by activation of cellular immunity by lymphocytes. The relationship between inflammatory biomarkers and PoAF has been demonstrated in many studies^[^^6^^]^. Therefore, PNI may be a risk factor for PoAF. Also PNI has been used to predict prognosis and poor outcome after myocardial infarction^[^^7^^]^, and in patients who underwent CABG^[^^8^^]^.

In this study, we aimed to investigate the importance of PNI and VAI in predicting PoAF, which occurs after CABG operations accompanied by cardiopulmonary bypass (CPB).

## METHODS

Patients who underwent isolated CABG with CBP in Bursa Yüksek İhtisas Training and Research Hospital between June 15 and October 15, 2019, were prospectively included in the study, which began after ethics approval was obtained. All patients signed an informed consent form prior to the operation. All procedures were performed in accordance with the Declaration of Helsinki. Preoperative demographic features, blood parameters, physical examination findings (height, weight, waist circumference [WC]), and operative (cross-clamp and CPB times) and postoperative data (PoAF status, hospital stay, intensive care hospitalization time) were recorded. Patients with known paroxysmal-persistent atrial fibrillation (AF), those using preoperative amiodarone, those with known systemic inflammatory disease, moderate to severe heart valve disease, a left atrium diameter ≥ 40 mm, creatinine values > 1.5 mg/dL, patients who underwent acute coronary syndrome in the past month, emergency operations, those who underwent perioperative myocardial infarction, and who had intra-aortic balloon insertion were excluded. After the implementation of exclusion criteria, 199 patients were included in the study. The primary endpoint of the study was defined as the development of intra-hospital PoAF. Patients who did not develop in-hospital PoAF were identified as Group 1, and those who did constituted Group 2.

### Calculation of VAI

Patients' heights and weights were measured while wearing a standard pre-surgical gown with a known weight. WC was measured from the midline between the iliac crest and the lower border of the lower rib while patients were standing. The blood parameter values were obtained from the preoperative routine tests and those of each patient were entered into the computer program. The following formulations were used for calculation^[^^5^^]^:

$$\begin{array}{c} \mathrm{Females}:\;\mathrm{VAI}\left(\frac{\mathrm{WC}}{36.58\;x\;\left(1.89\;x\;\mathrm{BMI}\right)}\right)x\left(\frac{\mathrm{TG}}{0.81}\right)x\left(\frac{1.52}{\mathrm{HDL}}\right)\\ \mathrm{Males}:\;\mathrm{VAI}=\left(\frac{\mathrm{WC}}{39.68\;x\;\left(1.88\;x\;\mathrm{BMI}\right)}\right)x\left(\frac{\mathrm{TG}}{1.03}\right)x\left(\frac{1.31}{\mathrm{HDL}}\right) \end{array}$$

Where:

WC: cm
BMI: kg/m^2^
Triglycerides (TG): mmol/L
High-density lipoprotein (HDL): mmol/L

### Calculation of PNI

The values obtained from the routine preoperative blood tests of the patients were used to calculate PNI with the formula below:

$$\mathrm{PNI}=\mathrm{serum}\;\mathrm{albu}\min\;\mathrm{level}\left(g/L\right)+\mathrm{total}\;\mathrm{lymphocyte}\;\mathrm{count}\left(10^{3}/µL\right)\;x\;5$$

### Postoperative Rhythm Monitoring

All patients were transferred to the cardiovascular surgery intensive care unit after the operation. During both inpatient and intensive care follow-ups, electrocardiography (ECG) monitoring was performed, and 12-lead ECG recordings were obtained daily. They were also obtained if patients reported complaints such as palpitations and sweating when they were not monitored in their beds. AF was defined as the absence of a P wave and the development of irregular ventricular rhythm before the QRS complex, and it was considered PoAF once it lasted over five minutes. Patients who developed PoAF were primarily treated with intravenous beta-blockers for rate control, and intravenous amiodarone was administered in patients who did not respond to beta-blocker therapy. Cardioversion was performed in patients with unstable hemodynamics after the abovementioned treatments. If the patient has been in AF for > 48 hours and hemodynamic instability has developed, electrical cardioversion was performed following transesophageal echocardiography and exclusion of left atrial thrombus.

### Statistical Analysis

In this study, statistical analyses were performed using the IBM Corp. Released 2012, IBM SPSS Statistics for Windows, Version 21.0, Armonk, NY: IBM Corp. statistical package program. The *P*-values obtained in the test results were considered significant when < 0.05. Mean and standard deviations were calculated for continuous and ordinal data using descriptive methods. Kolmogorov-Smirnov and Shapiro-Wilk tests were used for evaluation of normality distribution. Student's t-test and Mann-Whitney U test were used to assess normally and non-normally distributed data, respectively. Frequency and percentage analysis were conducted for nominal data, which were compared with Chi-square test. Multivariate regression analysis was performed to show the effects of PoAF risk factors (Model 1 with PNI and Model 2 with VAI). Receiver-operating characteristic (ROC) curve analysis was performed for VAI and PNI in terms of predicting PoAF and the areas under the curve (AUC) were calculated.

## RESULTS

General characteristics of the patients included in the study are presented in Table 1. PoAF developed in 55 (27.6%) patients (Group 2). The mean age of the 144 patients included in Group 1 and 55 patients in Group 2 were 56.9±8.7 and 64.3±10.2 years, respectively (*P*<0.001). The rates of hypertension (HT) and COPD were higher in Group 2 (*P*=0.021, *P*=0.017, respectively). The two groups were similar in terms of gender, coronary intervention history, diabetes mellitus, BMI, beta-blocker use, and angiotensin-converting enzyme inhibitor/angiotensin receptor blocker use.

**Table 1:** Patients' preoperative variables and demographic data.

Characteristics	Group 1	Group 2	*P*-value
	(n=144)	(n=55)	
Age (years), mean±SD	56.9±8.7	64.3±10.2	< 0.001^†^
Male gender, n (%)	113 (78.4)	45 (81.8)	0.547*
Previous PCI, n (%)	24 (16.6)	11 (20)	0.672*
Hypertension, n (%)	85 (59.2)	46 (83.6)	0.021*
COPD, n (%)	21 (14.5)	21 (38.1)	0.017*
BMI (kg/m^2^)	29.4±4.6	30.4±6.1	0.201^†^
Diabetes mellitus, n (%)	30 (20.8)	13 (23.6)	0.779*
Smoking, n (%)	33 (22.9)	16 (29)	0.614*
Beta-blocker use, n (%)	35 (24.3)	10 (18.1)	0.572*
ACE-I/ARB use, n (%)	44 (30.4)	15 (25.4)	0.718*
Ejection fraction (%), mean±SD	50.8±7.5	48.3±6.6	0.196^†^
Left atrial diameter (cm), mean±SD	3.5±0.3	3.7±0.3	0.228^†^
EuroSCORE II	1.6 (0.5-4.8)	1.8 (0.5-5.2)	0.189^‡^

† Student's *t*-test

* Chi-square test

‡ Mann-Whitney U test (data is expressed as median [interquartile range])

ACE-I=angiotensin-converting enzyme inhibitor; ARB=angiotensin receptor blocker; BMI=body mass index; COPD=chronic obstructive pulmonary disease; EuroSCORE II=European System for Cardiac Operative Risk Evaluation II; PCI=percutaneous coronary intervention; SD=standard deviation

Patients’ preoperative laboratory values and perioperative features are summarized in Table 2. There was no significant difference between the groups in terms of white blood cell, neutrophil, low-density lipoprotein cholesterol, HDL cholesterol, and albumin values. In Group 2, lymphocyte values and PNI were significantly lower (*P*=0.004, *P*=0.003, respectively), whereas TG values, C-reactive protein, and VAI were significantly higher (*P*=0.038, *P*=0.018, *P*<0.001, respectively). Among operative data, the numbers of distal anastomoses, total perfusion, and cross-clamp times were similar in both groups. Intensive care length of stay and hospitalization time were higher in the patient group who developed PoAF (*P*<0.001).

**Table 2:** Patients' preoperative laboratory values and perioperative variables.

Characteristics	Group 1	Group 2	*P*-value
	(n=144)	(n=55)	
Hemoglobin (g/dL)	14.1 (12.2-16)	13.5 (12.4-16.8)	0.284^‡^
White blood cell (10^3^/µL)	8 (6.5-9.9)	8 (6.5-10.7)	0.201^‡^
Neutrophil (10^3^/µL)	4.4 (3.5- 9.7)	4.7 (3.6-9.9)	0.267^‡^
Lymphocyte (10^3^/µL)	2.2 (0.8-3.9)	1.8 (0.7-3.8)	0.004^‡^
Creatinine (mg/dL)	1.1 (0.36-1.49)	1.2 (0.44-1.48)	0.556^‡^
LDL-C (mmol/L)	3.3 (2.3-4.6)	3.5 (3-4.8)	0.216^‡^
HDL-C (mmol/L)	1 (0.7-1.4)	1.1 (0.7-1.6)	0.196^‡^
Triglyceride (mmol/L)	2 (0.7-5.5)	2.4 (0.9-5.9)	0.038^‡^
C-reactive protein (mg/dL)	7.4 (1.6-38)	9.1 (1.9-40)	0.018^‡^
Albumin (g/L)	39.2 (35-53)	38.7 (34.4-54)	0.212^‡^
Free T3 (pg/mL)	2.8 (2.3-5.2)	3 (2.2-5.5)	0.283^‡^
Free T4 (ng/dL)	0.7 (0.5-1.2)	0.8 (0.5-1.3)	0.678^‡^
TSH (µIU/L)	1.3 (0.8-4)	1.4 (0.7-4.2)	0.317^‡^
VAI	3.5 (1.2-14.9)	5.2 (2.4-15)	<0.001^‡^
PNI	48 (35-69)	45 (34-58)	0.003^‡^
Distal anastomosis number, n	4 (1-6)	4 (2-6)	0.393^‡^
CCt (minutes)	67 (44-91)	72 (52-96)	0.214^‡^
TPt (minutes)	96 (70-130)	120 (93-138)	0.302^‡^
Total chest tube drainage (ml)	450 (250-1350)	500 (300-1400)	0.418^‡^
Inotropic support, n (%)	8 (5.5)	12 (21.8)	0.094*
ICU length of stay (days)	2.5±1	3.4±1.2	< 0.001^†^
Total hospital length of stay (days)	6.8±1.2	8.5±1.2	< 0.001^†^

† Student's *t*-test (data is expressed as mean±standard deviation)

‡ Mann-Whitney U test (data is expressed as median [interquartile range])

* Chi-square test

CCt=cross-clamp time; HDL-C=high-density lipoprotein cholesterol; ICU=intensive care unit; LDL-C=low-density lipoprotein cholesterol; PNI=prognostic nutritional index; TPt=total perfusion time; TSH=thyroid-stimulating hormone; VAI=visceral adiposity index

Logistic regression analysis was performed to reveal the factors affecting PoAF occurring in the hospital. In univariate analysis, PoAF was found to significantly correlate with age (odds ratio [OR]: 1.151, 95% confidence interval [CI]: 1.008-1.244, *P*<0.001), HT (OR: 0.824, CI: 0.756-0.978, *P*=0.026), COPD (OR: 0.653, CI: 0.312-0.776, *P*=0.023), lymphocyte counts (OR: 0.396, CI: 0.294-0.804, *P*=0.010), C-reactive protein (OR: 1.050, CI: 1.008-1.212, *P*=0.028), PNI (OR: 1.024, CI: 1.010-1.196, *P*=0.002), and VAI (OR: 1.412, CI: 1.186-1.697, *P*<0.001), and not correlate with TG levels. In multivariate analysis Model 1, age (OR: 1.084, CI: 1.010-1.176, *P*=0.009), COPD (OR: 0.798, CI: 0.664-0.928, *P*=0.048), and PNI (OR: 1.052, CI: 1.015-1.379, *P*=0.011) were determined as independent predictors for PoAF. In Model 2, age (OR: 1.078, CI: 1.008-1.194, *P*=0.012), lymphocyte counts (OR: 0.412, CI: 0.374-0.778, *P*=0.032), and VAI (OR: 1.516, CI: 1.314-2.154, *P*<0.001) were determined as independent predictors for PoAF.

**Fig. 1:**
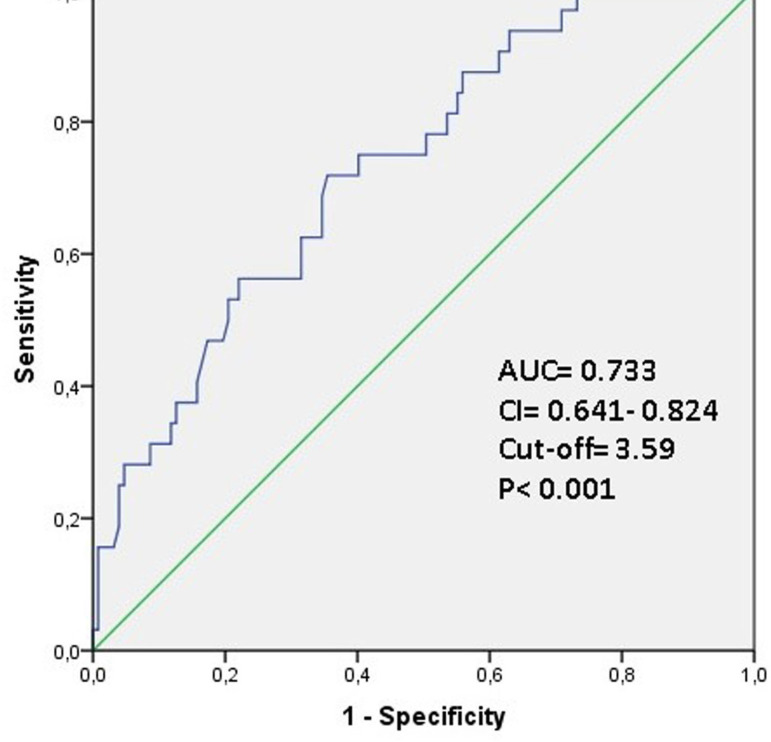
Data figure of the area under the curve (AUC), confidence interval (CI), and cutoff values in receiver-operating characteristic (ROC) curve analysis for visceral adiposity index (84.6% sensitivity, 53.1% specificity).

**Fig. 2:**
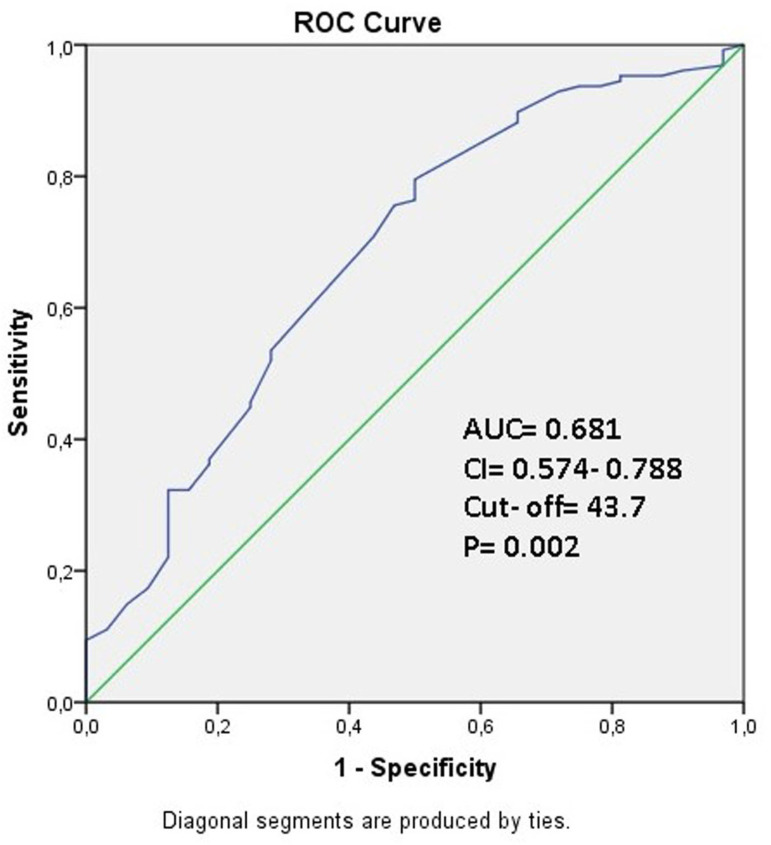
Data figure of the area under the curve (AUC), confidence interval (CI), and cutoff values in receiver-operating characteristic (ROC) curve analysis for prognostic nutritional index (76.4% sensitivity, 48.3% specificity).

ROC curve analysis revealed that the cutoff values for VAI ratio and PNI were 3.59 (AUC: 0.733, 95% CI: 0.641-0.824, *P*<0.001, 84.6% sensitivity and 53.1% specificity) (Figure 1) and 43.7 (AUC: 0.681, 95% CI: 0.574-0.788, *P*=0.002, 76.4% sensitivity and 48.3% specificity) (Figure 2).

## DISCUSSION

AF, which occurs after CABG, is undesired due to mortality, cerebrovascular events, heart failure, and the financial burden caused by prolonged hospitalization^[^^9^^]^. Many studies have been and are currently conducted to reveal possible risk factors for this condition. Although they have been extensively studied in other areas of medicine, in this prospective study, we explored the roles of PNI and VAI values in predicting PoAF after CABG operations and determined that low PNI and high VAI were independent predictors for PoAF.

Obesity has become one of the most important problems of our era, as humans switched to office-style living. Cardiovascular diseases are the leading problems caused by this situation. Obesity can also affect undesired outcomes such as PoAF after treatments like CABG. BMI is the most commonly used parameter as an indicator of obesity. In a meta-analysis conducted by Hernandez et al.^[^^3^^]^ in 2013, obesity (defined by BMI) was reported as a risk factor of PoAF, regardless of the type of cardiac surgery. In the meta-analysis of Aune et al.^[^^10^^]^ involving prospective studies on general population, BMI and WC were found to be associated with AF. However, in this study, AF was more frequent in the group with a BMI value of 20 kg/m^2^ compared to the group with BMI values between 22 and 24 kg/m^2^. In parallel with this finding, although obesity was found to affect the development of cardiovascular diseases in recent studies, it has been reported that overweight and mildly obese patients with cardiovascular disease have a better prognosis than low or normal weight patients^[^^11^^,^^12^^]^. Consequently, WC value, which is thought to be more valuable in the formation and prognosis of cardiovascular diseases, have been the subject of research. It is an important indicator of central obesity and has been shown to be a risk factor for AF^[^^13^^]^. In another study, it was found that central obesity may be more valuable than BMI in predicting PoAF after CABG^[^^14^^]^. In a retrospective study conducted by Girerd et al.^[^^15^^]^, WC of 102 cm and above was reported as an independent predictor for PoAF after CABG in men (OR: 1.40, *P*=0.04).

VAI, calculated by formulating parameters such as WC, BMI, TG, and HDL, is a valuable indicator of visceral fattening, and calculated with separate formulas for women and men. In a study conducted on general population, the rate of visceral adipose tissue was found to be more predictive for cardiovascular risks compared to BMI and WC^[^^16^^]^. In another study of 1498 patients, VAI was found to be an independent predictor for cardiovascular and cerebrovascular events, and VAI was reported as a more sensitive and specific predictor than its components^[^^5^^]^. Increased VAI values were also associated with cardiovascular events in a large population case-control study^[^^17^^]^. In light of all this information, we also determined VAI as an independent predictor in predicting PoAF after isolated CABG.

Malnutrition is an important public health problem in developed countries. PNI is a valuable malnutrition parameter obtained by adding five times the lymphocyte counts to the serum albumin value. Hypoalbuminemia is closely related to malnutrition and a good predictor for surgical risk. It has also been shown as an independent predictor of many cardiovascular diseases, including heart failure, coronary artery disease, and stroke. The importance of albumin stems from its anti-inflammatory, antioxidant, and anticoagulant properties and its effects on osmotic pressure^[^^18^^]^. Lymphopenia is associated with major adverse events in atherosclerotic cardiovascular diseases^[^^19^^]^. The importance of high neutrophil/lymphocyte ratio in predicting prognosis after CABG is known and many studies are available on this subject^[^^20^^]^. The increase of this ratio is due to decreased lymphocyte count along with elevated neutrophil count. As a result, the number of 5x lymphocytes used in PNI calculation appears to be a valuable parameter.

**Table 3:** Logistic regression analysis to identify factors affecting postoperative atrial fibrillation.

Variables	Univariate analysis			Multivariate analysis		
	*P*-value	Exp (B)	95% CI	*P*-value	Exp (B)	95% CI
		Odds ratio	Lower-upper		Odds ratio	Lower-upper
Age	< 0.001	1.151	1.008-1.244	0.009^a^	1.084^a^	1.010-1-176^a^
				0.012^b^	1.078^b^	1.008-1.194^b^
Hypertension	0.026	0.824	0.756-0.978	0.096^a^	0.876^a^	0.794-1.100^a^
				0.112^b^	0.914^b^	0.678-1.145^b^
COPD	0.023	0.653	0.312-0.776	0.048^a^	0.798^a^	0.664-0.928^a^
				0.114^b^	0.678^b^	0.479-1.217^b^
Triglyceride	0.064	0.897	0.674-1.010	--	--	--
Lymphocyte	0.010	0.396	0.294-0.804	--	--	--
				0.032^b^	0.412^b^	0.374-0.778^b^
CRP	0.028	1.050	1.008-1.212	0.108^a^	1.090^a^	0.928-1.234^a^
				0.176^b^	1.078^b^	0.879-1.194^b^
PNI	0.002	1.024	1.010-1.196	0.011^a^	1.052^a^	1.015-1.379^a^
VAI	< 0.001	1.412	1.186-1.697	< 0.001^b^	1.516^b^	1.314-2.154^b^

a Multivariate analysis Model 1 (the goodness of fit of the model was confirmed by a *P*-value of 0.695 in the Hosmer-Lemeshow test)

b Multivariate analysis Model 2 (the goodness of fit of the model was confirmed by a *P*-value of 0.741 in the Hosmer-Lemeshow test)

CI=confidence interval; COPD=chronic obstructive pulmonary disease; CRP=C-reactive protein; PNI=prognostic nutritional index; VAI=visceral adiposity index

There are studies showing the prognostic significance of PNI value, an easily and rapidly calculable parameter which yields valuable information about nutritional status, in malignancy and hemodialysis patients^[^^21^^,^^22^^]^. In infants under the age of 18 months who underwent open heart surgery, it has been found to be associated with prolonged intensive care hospitalizations and poor postoperative outcome^[^^23^^]^. Yost et al.^[^^24^^]^ found it effective in predicting one-year survival in patients with a left ventricular support device. In that study, its effect on the development of early right ventricular failure following the operation was also reported.

The most recent study investigating the importance of PNI in patients undergoing cardiac surgery with CPB was published in the last months of 2019, in which 374 patients were retrospectively included. PNI cutoff value was determined as 46.13, and patients were divided into two groups as those with a high (< 46.13) and low risk (> 46.13). Early mortality and morbidity were significantly higher in the high-risk group (9.0% *vs*. 2.9%: *P*=.02, 58.0% *vs*. 42.0%: *P*=.01, respectively). In addition, multivariate analysis showed that PNI was an independent predictor of early mortality. Based on these results, the authors suggested that the value of PNI, which can be easily calculated preoperatively, is a valuable parameter in predicting early results in patients undergoing cardiac surgery with CPB^[^^25^^]^. In our study, we determined PNI as an independent predictor in predicting PoAF, one of the important causes of morbidity after CABG. However, VAI was more predictive than PNI.

Advanced age is a risk factor for PoAF. There is an increase in the AF incidence due to structural shifts in the heart and atrial fibrosis related to the advanced age. Also, COPD is a known predictor of PoAF^[^^6^^]^. In our current study, advanced age was found to be an independent predictor of PoAF in multivariate Model 1 and Model 2. And COPD was found to be an independent predictor in Model 1.

### Limitations

The fact that this is a single center study with a low sample size is the most important limitation. Although PNI and VAI had good sensitivity, they had low specificity. Further prospective studies with a larger number of patients are required.

## CONCLUSION

PoAF, which occurs after CABG, is particularly important due to possible mortality and morbidity outcomes. Predicting the possible risk factors of this condition is highly valuable for cardiac surgeons, and many studies have been done in this field. In this study, we determined that low PNI, a simply calculable and cheap parameter, along with high VAI were risk factors for PoAF. Physical examination evaluations of height, weight, and body surface area are routinely performed before cardiac surgery. However, WC measurement is not a routine assessment for cardiac surgeons. WC may become a parameter in our routine examination if our research is supported by larger scale new studies.

**Table:** 

Authors' roles & responsibilities	
ME	Substantial contributions to the conception or design of the work; or the acquisition, analysis, or interpretation of data for the work; agreement to be accountable for all aspects of the work in ensuring that questions related to the accuracy or integrity of any part of the work are appropriately investigated and resolved; drafting the work or revising it critically for important intellectual content; final approval of the version to be published
KKO	Substantial contributions to the conception or design of the work, or the acquisition, analysis, or interpretation of data for the work; final approval of the version to be published
MS	Substantial contributions to the conception or design of the work; or the acquisition, analysis, or interpretation of data for the work; final approval of the version to be published
OG	Substantial contributions to the conception or design of the work; or the acquisition, analysis, or interpretation of data for the work; final approval of the version to be published
SY	Drafting the work or revising it critically for important intellectual content; final approval of the version to be published
AFO	Drafting the work or revising it critically for important intellectual content; final approval of the version to be published
